# The Functional Psychotherapy Approach: A Process-Outcome Multiple Case Study

**DOI:** 10.3389/fpsyg.2021.727497

**Published:** 2022-01-07

**Authors:** Diego Rocco, Luca Rizzi, Gaia Dell’Arciprete, Raffaella Perrella

**Affiliations:** ^1^Department of Developmental and Social Psychology, University of Padua, Padua, Italy; ^2^Associazione Centro di Psicologia e Psicoterapia Funzionale, Istituto SIF, Padua, Italy; ^3^Department of Psychology, University of Campania “Luigi Vanvitelli”, Caserta, Italy

**Keywords:** functional psychotherapy, psychotherapy outcome, psychotherapy process, multiple case study, process-outcome

## Abstract

**Objective:** The present work aims to conduct the first naturalistic empirical investigation of the process and outcome assessment of functional psychotherapy (FP) treatment. The FP model of psychotherapy is rooted in psychoanalysis and integrates the verbal communication approach founded on transference and countertransference dynamics with the analysis of bodily processes.

**Method:** The study sample included ten patients recruited on a voluntary basis and treated by clinicians in their private practices. Each patient received FP with an average duration of 40 h (min 35 and max 42). Therapies had weekly sessions, were audio-recorded with the patient’s written consent, and lasted for an average of 10 months (min 9 and max 12). Outcome and process tools included the Minnesota Multiphasic Personality Inventory-2 (MMPI-2) and the Luborsky’s the Core Conflictual Relationship Theme (CCRT), used to assess therapeutic benefit, and the Metacognition Assessment Scale (MAS) and the Italian Discourse Attributes Analysis Program (IDAAP) system, used to evaluate therapeutic benefit and process. The MMPI-2 was used also in the follow-up assessment.

**Results:** Results show that FP had a positive therapeutic outcome on the patients assessed in this study, and that the therapeutic benefits were maintained over time. Some specific features of the FP approach were found to contribute more than others to the observed therapeutic benefits.

**Conclusion:** The current investigation constitutes a first step toward assessment of the therapeutic effectiveness of FP. Future developments should apply the methodology to a larger sample, possibly introducing different methodologies to enable detection of specific bodily oriented processes and techniques.

## Introduction

During daily clinical experiences, after meeting a client for the first clinical interview, it is necessary to evaluate what kind of treatments – if any – could be suitable for their specific needs or disorders.

In order to make reliable and ethical recommendations, clinicians as well as the psychotherapy community, should gather information on which intervention model has been shown to be effective in treating a specific disorder (Leichsenring, 2009). One of the origins of the difficulty of assessing the efficacy of a psychotherapy model lies within the origin of psychotherapy itself (Migone, 2006). In an attempt to create the best scientific assessment conditions, several methodological proposals have been offered over the years. The most common scientific design, the randomized controlled trial (RCT; see Shadish et al., 2002), is a methodology that has its roots in medical/pharmacological treatments and intends to systematically control all relevant variables, other than the treatment itself, that could influence the therapeutic outcome. This approach represents the “gold standard” for demonstrating the efficacy of a treatment, since it must be conducted under strictly controlled experimental conditions (Gelo et al., 2010).

In the view of many scholars (e.g., Roth and Parry, 1997; Persons and Silberschatz, 1998; Westen et al., 2004), the RTC approach also presents some problems, mainly concerning the strict limitations that the methodology imposes. From these limitations stems the question as to whether RCTs are sufficiently representative of “normal” daily clinical practice. In such a setting, not every external condition can be systematically controlled and clinicians follow “real” patients, that is, patients that do not represent a “pure” form of a specific disorder. This is why, even if a treatment has an empirical support based on RCTs’ results, its validity from the research setting to the daily clinical setting should not be taken for granted. The transposition from the efficacy (in the RCTs) to the effectiveness (in daily clinical activity) of a treatment represents an important issue, that has to do with the feasibility, generalizability, and cost of therapeutic procedures (Comer and Kendall, 2013). An alternative to RCTs are naturalistic studies, that is, studies conducted under the same conditions of clinical practice with regards to patients, therapists, and treatments (Shadish et al., 2000). These *quasi-experimental* research designs (Shadish et al., 2002; Leichsenring, 2009) include groups of patients which are not selected on the basis of the researcher’s criteria (as for RCTs), but rather try to mimic as much as possible the conditions and characteristics of patients encountered during clinical practice. It is important to underline that several studies have provided evidence that naturalistic studies do not overestimate effect sizes when compared to RCTs (Benson and Hartz, 2000; Shadish et al., 2000), thus they represent a research methodology equally valid and reliable.

In both RCTs and quasi-experimental methodologies, in order to gather reliable evidence on the efficacy/effectiveness of a treatment, some specific conditions are required. First of all, it is necessary to identify the techniques used within the treatment itself, with the aim of allowing the recognition of *that* specific treatment among the others on the basis of *its* specific technique. To achieve this goal, a system of treatment adherence judgment should be followed. Secondly, in order to verify the treatment’s broader efficacy, the improvement in therapeutic outcome should be evaluated on more than just a single dimension (e.g., symptom improvement), considering also wider dimensions (e.g., general functioning, personality structure changes; Comer and Kendall, 2004; Gelo et al., 2010; Rocco et al., 2021a). Other aspects that should not be ignored are the collection of follow-up data that enable assessment of longer-term therapeutic benefit, and treatment duration. Moreover, it may be best to have at disposal therapeutic outcomes deriving from long-term treatments, instead of focusing only on short-term treatments.

It is following these and others methodological aspects that a treatment starts to be considered, after a long iterative procedure, as part of the Empirically Supported Treatments (Chambless, 1995). In fact, it is necessary to accumulate a great deal of evidence on evaluated outcomes before it can be concluded that a treatment is effective (Comer and Kendall, 2013).

Given these historical and methodological premises, in the current research we aimed to perform, within a naturalistic empirical framework, the first explorative assessment of the therapeutic outcomes of functional psychotherapy (FP)^1^ intervention treatment (Rispoli, 1993, 1999, 2008, 2016) through a process-outcome multiple case study methodology (for further examples of this research design, see Burke et al., 1998; Semerari et al., 2005; Dimaggio et al., 2009; Carcione et al., 2011). This specific model of psychotherapy has its underpinnings in psychoanalysis, more precisely in Reich (1933), Lowen (1958), and Boyesen (1986) and, on the basis of its theoretical and technical foundations, can be included, according to Gabbard’s classification (Gabbard, 2004), in the psychodynamic psychotherapy treatments, since it operates within the interpretive–supportive continuum, choosing a more interpretive or supportive intervention according to the patient’s needs. FP is also included in the long-term psychodynamic psychotherapies, since it usually has more than 24 sessions or lasts more than 6 months (Gabbard, 2004).

In the second part of our research, we aimed to deepen some of the features of the FP process in order to identify the specificity of this kind of intervention model, that is characterized by a bodily/corporeal technical approach. In this regard, we chose methodologies based on the analysis of session transcripts which, within a multiple case-study methodology, can be simultaneously used as parameters to analyze the process features and outcome measurements. We expect on the one hand that the techniques of the FP approach, specifically focused on bodily processes, will foster therapeutic processes characterized by abrupt patient contact with emotional aspects, since the defensive system should be more easily bypassed by the technique itself. On the other hand, since metacognitive impairments are usually present in case of psychopathology, we expect that there will be a slow yet constant increase in patients’ metacognitive capacity of self-reflexivity during psychotherapy sessions, given the clinical nature of our sample.

The third aspect we aimed to analyze, based on this multi-perspective and multi-instrumental research approach, was the relationship between outcomes of the therapies and the underlying processes. Specifically, we aimed to verify in each of the single therapies we considered which aspects of the FP approach promote the development of a positive, negative or null outcome. In this regard, we adopted the process/outcome- research approach (Crits-Christoph et al., 2013) and explored the role of both processes and micro-processes. We expect that if the patient’s contact with emotions fostered by FP techniques is complemented by an adequate level of elaboration of their same emotional experience, this will be associated with improved therapeutic outcomes. Concerning the relationship between patients’ metacognitive capacity and therapeutic outcome, we expected that when self-reflexivity has a slow but constant increase, the therapeutic outcome will be more positive. Lastly, we expect that when both aspects are present to a higher degree (i.e., patient contact with their emotions, complemented by an adequate level of elaboration, and an increase in self-reflexivity), more positive therapeutic outcomes will be observed.

Given its features, the multiple case study methodology should allow us to create a link between FP practice and empirical research, thereby enabling them to reciprocally use their findings and evidence to make progress in psychotherapy effectiveness (Iwakabe and Gazzola, 2009).

## Materials and Methods

### Enrollment

Sample enrollment conformed to the Declaration of Helsinki (World Medical Association [WMA], 2013). The ten patients considered in this study were met by the clinicians in their private practice and joined the study on a voluntary basis. Before taking part in the research, they were asked to read and approve a written informed consent document. Participants were briefed on privacy, the use of data and were informed of their right to withdraw at any time, with no repercussions. The informed consent specified that, during the psychotherapeutic intervention, the psychotherapists would engage the patients in techniques that involve movements (such as walking, opening arms, and kicking hard, etc.) and direct touch of the patients’ body (such as massaging, feeling the breathing rhythm, and playing, etc.); it was explained that these techniques help patients to develop, within the therapeutic relationship, an appropriate non-verbal behavior, body awareness and other Basic Experiences of Self (BES; see “Treatment and adherence” paragraph below). Complete anonymity was guaranteed to all participants. To be eligible for the study, adult participants were required to take part in a FP and not present psychotic symptoms or syndromes. Demographic and baseline characteristic are reported in Table 1.

**TABLE 1:** Demographic and baseline characteristics.

	All patients (*N* = 10)
**Age (years)**	
Mean ± SD	35.7 ± 7.32
Min, max	29,47
**Gender, *n* (%)**	
Female	7 (70.00%)
Male	3 (30.00%)
**Occupation *n* (%)**	
Employee	6 (60.0%)
Self-employee	3 (30.0%)
Unemployed	1 (10.0%)
**Marital status *n* (%)**	
Single	3 (30.0%)
Married	5 (50.0%)
Cohabitant	1 (10.0%)
Engaged	1 (10.0%)
**Diagnosis *n* (%)**	
Generalized anxiety disorder	4 (40.0%)
Social anxiety disorder	1 (10.0%)
Paranoid personality disorder	1 (10.0%)
Antisocial personality disorder	1 (10.0%)
Borderline personality disorder	2 (20.0%)
Obsessive-compulsive personality disorder	1 (10.0%)

### The Psychotherapists

The psychotherapies were carried out by 8 psychotherapists (3 women and 5 men), with a mean age of 45.9 years (from 32 to 73 years old), who had more than 5 years of clinical and diagnostic experience and were specifically trained for the use of the FP model.

### Treatment and Adherence

Functional psychotherapy lays its foundations in psychoanalysis, more precisely in the work of Wilhelm Reich, one of Freud’s students. Reich (1942) integrated the two main psychoanalytic ways to access the patient’s psyche – verbal communication and transference/countertransference – with the analysis of bodily processes. Reich claimed that the body is not an obstacle to therapy but, instead, can play an important role, as mental processes do not fully control a person’s behavior and bodily processes influence thoughts and mental representations. Lost processes of symbolization, that otherwise would have remained undetected, might be recovered through the body. According to Reich, one must think of a sort of “body memory,” a peripheral memory consisting of permanent traces of past experiences, which include repetitive and habitual postures, permanent alteration of the perceptual thresholds, chronic changes in basic muscle tone and movements sculpted over time.

In the bodily intervention, developed by Reich’s supporters, the body became an instrument for psychotherapists to assess patients’ mental processes and change them. The FP approach was developed in Italy during the 1980s, within the context of body psychotherapies, by Luciano Rispoli (1993, 2008, 2016). The novelty of this approach, compared to the other body approaches, is that FP is based on Functionalism (James, 1890) and takes advantage of neuroscience and psychoneuroendocrinology findings to understand the bond between body and mind. Psychoneuroimmunology (PNEI; Ader, 2000) involves studying interactions between psychological, neural, endocrine, and immunological processes. PNEI supports the idea that the organism is a self-regulated network governed by four systems: the physiological mechanisms proper of human psyche, the neurological system (namely, the limbic system and hypothalamus in primis), the endocrine system (e.g., hypophysis and receptor glands) and the immunesystem. The integrated self-regulation network connecting these systems is implicated in the psychosomatic homeostasis of the organisms in response to endogenous and exogenous stimuli (Lekha, 2020). According to PNEI, FP intervenes on these systems and their integrated network.

Firmly rooted in contemporary science, FP is designed to be suitable for many clinical and personality disorders (Rispoli, 2016) and highlights the possibility of inducing measurable changes on the cognitive, emotional and psychological systems through a therapeutic relationship based on both verbal and non-verbal communication (Reich, 1942; Lowen, 1958; Totton and Edmondson, 2007; Klopstech, 2008; Perrella, 2017). According to this approach, the body “talks” through periods of silence, voice inflections, gestures, postures and its physiologic functioning (temperature, rigidity, hormonal release, etc.). Both the therapist and the patient’s bodies are considered as objects to be analyzed, and the therapist’s bodily reactions during the clinical sessions are part of the “expanded countertransference” (Rispoli, 2008).

The FP body techniques serve specific therapeutic functions and are organized upon their experiential aim. Tthe functional psychotherapist can apply a specific array of techniques to foster the development of certain resources for managing stress, cultivating wellbeing and maintaining a good mental health (Maniaci et al., 2018). The experiential aims are defined by the concept of basic experiences of the self (BES), which are those specific experiences necessary to satisfy human basic needs (Rispoli, 2008).

The functional therapist promotes the BES by leading the client through a wide range of experiential exercises, lasting from a few minutes to half an hour. The experiential techniques used in the FP involve body and physical contact to influence the whole person. For instance, the touch-massage (Smith, 1998; Rizzi et al., 2011; Field, 2016; Arnold et al., 2020) works on different systems in different ways: it affects the physiological and postural system through the technical aspects of massage and manipulation, the emotional system through the awareness of the use of touch, and the cognitive system through the use of images and words recalling memories. Indeed, touch strengthens the therapeutic relationship and contributes to create a warm and safe context.

Table 2 reports two examples of BES, along with the description of some specific techniques which can be adopted to foster them, results to be expected in case of successful application and, lastly, clinical examples drawn from therapies belonging to our clinical sample.

**TABLE 2:** Examples of BES, FP techniques, general observations, and clinical extracts from our sample’s therapies.

BES	Technique			General observations	Example
	
	Touch	Movement	Imaginary		
Sensations	Hand-arm massage (Rispoli, 1999; Rizzi et al., 2020) – The therapist invites the patient to lay down, closing his eyes and breathing deeply. Then, with a gentle, slow and deep touch, the therapist massages both patient’s arms and hands	Standing, loosen the head and the neck (Rispoli, 1999) – The therapist invites the patient to stand up with legs slightly apart. The patient breathes deeply and closes his eyes. Then the therapist invites him to gently move his head in any direction, accompanying the movement with his breathing	Imagine the breathing wave (Rispoli, 1999; Casetta et al., 2018) – The therapist invites the patient to lay down, close the eyes and breathe deeply. Then he suggests to the patient to imagine his breathing as a sea wave, coming and going, rhythmically rocking him	Working on this BES some patients can’t actually feel their body, because they are used to “think” the body and the feelings. The therapist can recognize this skill lack in the wrong use of the breath (it’s not coordinated with the movement) or in the patient reporting, at the end of the experience, personal reflections such as “I felt as in vacation” or “I felt I’m not good at doing this” instead of “I felt tension, or a sense of heaviness, warmth, …”	After the hand-arm massage, E. reports feeling his hands getting warmer, and she recalls the memory of baking with her grandmother. She is more relaxed, her voice is lower and softer. With the therapist she decides to try repeating the experience, asking his partner to massage her
Vitality	Tossing around (Rispoli, 1999) – The therapist invites the patient to lay down, closing his eyes and breathing deeply. Then the therapist starts tossing around his patient, hitting him gently but quickly, in a joking way, accompanying what he is doing with funny suggestions and ringing voice	The body follows the arms (Rispoli, 1999) – The therapist invites the patient to stand up with legs slightly apart. The patient breathes deeply and closes his eyes. Then the therapist invites him to move his hands and arms until the arms drag the whole body behind them. So, the patient starts turning, jumping, twisting, leaping	Imaging sand dunes (Rispoli, 1999) – The therapist invites the patient to lay down, close the eyes and breathe deeply. Then he suggests to the patient to imagine himself standing on the top of a sand dune and to start running down, laughing, enjoying, letting go any thought and worry, as if he were a child	Working on this BES some patients feel rigid and worried, and aren’t able to have fun, feel the vitality and let go of their need for control. For some people these techniques can be very challenging	After the Body follows the arms, U. reported she had fun, even if initially she noticed her judgments about what she was doing, thinking “you look like a stupid.” She said that after shouting up her thoughts she just felt free to move as when she was a child. When talking, her voice was more ringing, her shoulders were straight and her eyes were smiling

Due to social distancing regulations during the Covid-19 pandemic, functional therapists excluded techniques based on touch, using instead those based on movement and imaginary, in order to ensure that an appropriate distance from patients was maintained, in accordance with health and safety regulations. The setting for the FP is structured to allow flexibility in the proximity between therapist and patient. The room must be wide enough to make it possible to move and to walk – in other words, to freely express each emotion through the body. Usually, the therapeutic setting consists of a 2 × 2 m soft surface (a bed, or a pile of mattresses) where the patients can lie down, and it allows the therapist to sit close to the patients if they need to touch them or adjust their postures. The therapist can otherwise stay quite distant (1–2 m) if they plan to use only their voice to accompany the patient’s through a visualization or a movement sequence aimed to listen to the body’s feelings. Techniques are designed to relax the patient and enable them to feel and express strength or anger, as well as to recall memories (Ogden and Fisher, 2015). Near the bed, there are always some pillows and a blanket, sometimes used in play experiences, but also useful to create a comfortable place. The room is also furnished with a desk and two chairs. There should not be many cabinets or other furniture to avoid dangerous corners and to prevent the setting from becoming overly packed.

The weekly 1-h session usually begins with a welcome where the client is asked to share their dreams, the important events of the week or talk about what happened during the previous session. Then, based on the client’s needs or on the therapeutic project, the therapist leads the patient to a defined BES using one or more functional techniques, as described in the functional techniques book by Rispoli (1999, 2016). This body work can last from 20 to 40 min. The therapist leads the patient in the BES by the modulation of paraverbal aspects of his/her voice (Galvani, 2019) and words, possibly using a musical background to enhance the emotional tone of the words content and to elicit images or memories coherent with the BES (Casetta and Rizzi, 2018). This experiential section usually ends with a reflection time, lasting a few minutes, during which the patient can silently observe their feelings, memories and physical sensations. Then, in the last part of the session, patients share their experience with the therapist, who can either just listen to the patient’s point of view or contextualize it within the therapeutic relationship or other situations outside the therapeutic setting.

In this research, the ten psychotherapies were held in a private setting and had an average duration of 40 h (min 35 and max 42). Therapies conducted weekly sessions, audio-recorded with the patients’ written consent, over an average period of 10 months (from 9 to 12).

The adherence to the FP approach was assured by the analysis of sessions’ verbatim transcription done by psychotherapists specialized in FP model. The presence of FP specific techniques, such as “sequence on tenderness,” “touching pain,” “cradling the patient’s legs,” “cradled from behind our shoulders,” “pushing with the arms,” and “neck and shoulder massage” (Rispoli, 2008), give the judges the possibility to verify without doubts the adherence to the model.

### Measures

We chose the following tools to evaluate both the process and the outcome of therapies since, on one hand, they are all already validated and widely used in literature (Dimaggio et al., 2007; Carcione et al., 2011; Salvatore et al., 2012; Perrella et al., 2013; Gennaro et al., 2017) and, on the other hand, they detect different and relevant aspects of the therapy, thus taken together they provide an exhaustive and comprehensive view of the whole clinical experience.

### Tools for Outcome Evaluation

#### The Minnesota Multiphasic Personality Inventory-2

The Minnesota Multiphasic Personality Inventory-2 (MMPI-2; Pancheri and Sirigatti, 2002) is a clinical assessment tool, consisting of 567 true-false questions, used by psychologists to diagnose mental health disorders. It was administered by the psychotherapist themselves, and then assessed and interpreted by two independent judges who received specific training on the use of MMPI-2. The MMPI-2 is composed of over 120 scales, many of which developed to evaluate clinical problems. The qualitative analysis of significant factors highlights that the “content scales” describe dimensions which are clinically useful to understand the patient’s behavioral and symptomatic manifestations; moreover, they identify the specific personality traits that explain their own way of thinking and behaving. On the other hand, the “clinical scales” describe peculiar psychopathological manifestations of the patient’s condition in the hic et nunc.

The results of the MMPI-2 must be considered as an interpretative method that may be useful in generating hypotheses about specific functioning areas (Donà et al., 2006). The tool can also help to collect information significant in formulating diagnostic hypotheses, given that high scores in clinical and contents scales usually reflect the typical symptomatology of DSM-5′s psychopathologies (American Psychiatric Association [APA], 2013).

#### The Core Conflictual Relationship Theme

The importance of detecting the recurrent aspects of patient’s interactions with other people, as expression of their inner intrapsychic conflicts, persuaded Luborsky to create a method to identify them. The result was the Core Conflictual Relationship Theme (CCRT; Luborsky and Crits-Christoph, 1998) methodology, that he conceived as an operationalization of the transference.

A patient’s CCRT, as mentioned above, represents the recurring way by which the patient interacts (or expects to interact) with others. Comparing the therapy’s initial and final CCRT allows one to appreciate the changes obtained in the patient’s methods of interaction at an intrapsychic level, starting from their manifestation at the interpersonal/relationship level. For this reason, it constitutes a method to detect the treatment outcome.

To determine the patient’s CCRT, the methodology – which is applied to therapy’s verbatim transcriptions – foresees two different evaluation phases. The first consists of collecting the patient’s interpersonal narratives (relational episode; RE) of their relationship with people, including the therapist and the self, referring to any period of their life. In the second phase different judges examine the selected set of RE in order to identify the three components of the CCRT specified by Luborsky: wishes, needs or intentions (W), responses from others (RO), and responses of self (RS). After some additional methodological steps, it is possible to come to the final classification of the components, called “standard category cluster,” which includes eight empirically derived categories for each component. The evaluation of the most recurrent components makes it possible to identify the CCRT, distinguishing between positive components (e.g., the RO, “the others like me”) and negative components (e.g., the RO “the others are rejecting and opposite”).

### Tools for the Therapeutic Process Assessment

#### Metacognition Assessment Scale

The Metacognition Assessment Scale (MAS; Carcione et al., 1997) is a tool that provides a quantitative estimation of changes in the metacognitive capacity in psychotherapy. It can easily be applied to transcripts of psychotherapy sessions and includes dichotomous items with yes/no answers, where “yes” demonstrates an effective and positive reaction and “no” reflects disappointment. The value of the MAS dwells in its modular methodology, which divides the construct into three sub-capacities: self-reflexivity, understanding the decentralization of the mind of others and mastery (every sub-capacity in its turn is additionally partitioned into extra subfunctions).

Different types of metacognitive deficits influence the patient’s clinical situation and the therapeutic relationship. However, the metacognitive functions consist of a set of connected yet semi-independent processes, thus each one can be harmed independently from the others. Moreover, there is evidence that some types of disorders are characterized by a specific impairment in self-reflexivity (Semerari et al., 2003, 2005, 2007; Dimaggio et al., 2007; Carcione et al., 2008, 2011); therefore, self-reflexivity is the only metacognitive function assessed in this study. Self-reflexivity (which is the subject’s ability to represent mental cognitions and perform heuristic cognitive procedures on their psychological working) comprises nine sub-capacities, with dichotomous qualities, that indicate the following variables: A1, A2 – essential requirements (the ability to perceive one’s own psychological states as autonomous); A3, A4 – characterization, (the ability to discriminate between cognitive and emotional aspects in one’s own inner states); A5, A6 – differentiation between mental portrayals and outside world; A7 – ability to construct relations between variables to clarify the purposes behind one’s own conduct; A8, A9 – integration (the ability to coordinate cognitive and emotional aspects into a cognizant verbal structure). When utilizing the MAS, the researcher should work on the recorded psychotherapy sessions by firstly dating them, then reading them and then dividing them into text units, which are pieces of the patient’s discourse included between two remarks; once the transcript is entirely divided into units, the researcher should pick, for each one of them, firstly the segment of the scale (in our investigation, self-reflexivity), secondly the function (e.g., integration) and, lastly, the item (e.g., A5). The latter item is scored as either success (A5 yes) or disappointment (A5 no) (Carcione et al., 1997; Semerari et al., 2003).

We assumed, based on previous studies and recent reflections (Perrella et al., 2013; Semerari et al., 2014) that a deficit or an excess in sub-functions can be detected more easily in some sub-functions of self-reflexivity rather than in others. In this work we aimed to deepen the relationship between metacognitive evolution and the outcome of clinical treatments.

#### The Referential Activity and the Italian Discourse Attributes Analysis Program

With the aim to create a method to analyze the therapeutic process, Wilma Bucci has developed the Multiple Code Theory (MCT; Bucci, 1985, 1997, 1999, 2021), a model of the cognitive/emotional functioning of the human mind that creates a bridge between psychoanalysis and cognitive psychology. The MCT considers three ways of processing information: subsymbolic (analogic processing on continuous dimensions), symbolic non-verbal (discrete, specific imagery or analogic patterns) and symbolic verbal (words; for the characteristics of these three systems of information processing see Bucci, 1997, 2021). The integration of these three information processing formats is up to the referential process, a complex cognitive function that should be activated during psychotherapy sessions with the help of the therapist. The referential process allows the patient to integrate the connections between experiences and words which were previously dissociated and, therefore, created a fertile ground for psychopathology. The detection of the referential activity is possible thanks to the Italian Discourse Attributes Analysis Program system (IDAAP; Mariani et al., 2013; Rocco et al., 2013), a computer program that includes five dictionaries concerning different scales, each one detecting specific aspects of the referential process. The IDAAP reads texts, compares them word by word through its dictionaries, attributes a score for every dictionary to each speaker, for each turn of speech, for the whole transcript of each session, and then calculates a weighted average mean of the dictionaries scores. Each dictionary produces scores for one or more scale. With the IDAAP it is possible to obtain mean scores for both a micro and macro analysis of sessions, as well as an assessment of the extent of the referential process.

In this work we considered, among all the available scales (for their complete description of which, see Mariani et al., 2013), three scales, described below, that are strongly informative of the underlying referential process activated during the psychotherapy process.

The first is the Weighted Referential Activity Dictionary (WRAD), which gives information on the mean referential activity, as in the ability to use the symbolizing function to translate visceral, relational and emotional experiences in words (Bucci, 1997, 2021; Mariani et al., 2013). The values of this scale range from 0 to 1, with a neutral value of 0.5, above which the referential activity can be considered high; thus, the higher the WRAD values, the higher the symbolizing function.

The second scale is the Weighted Reflection and Reorganization List (WRRL), which concerns “the degree to which the speaker is trying to recognize and understand the emotional significance of an event or set of events in their own or someone else’s life, or in a dream or fantasy” (Negri et al., 2019, p. 76). The WWRL must not be considered an abstract reflection, but rather the ability to be involved in the emotional experience while reflecting on it. The values of this scale range from 0 to 1, with a neutral value of 0.54, above which the reflection and reorganization activity can be considered high; thus, the higher the WRRL values, the higher the personal elaboration and understanding of emotional significance of events.

The third and last scale is the Reflection REF/WRAD covariation, which considers the relationship between the REF scale (that contains words concerning how people think and communicate their thoughts) and the WRAD dictionary, and therefore is a measure of the quality of the therapeutic elaboration that the patient is developing. Negative values of this covariation indicate the extent to which the speaker is able to separate the two functions, such as telling a story (characterized by high WRAD) and reflecting on it (characterized by high REF); negative values of this covariation indicate a greater separation between the two processes, that in literature is related to a clinical judgment of effectiveness (Bucci and Maskit, 2007; Mariani et al., 2013).

#### Procedure

The patients, at their first contact with a psychotherapist, were asked to complete the MMPI-2. They were asked to complete it also at the end of the treatment and at follow-up, which took place after an average of 18 months (from 12 to 24 months).

Four couples of consecutive sessions, the first at the beginning of the therapy and then a couple every 3 months, were entirely transcribed. This consecutiveness criterion was chosen to reduce the probability of a random error, in other words the possibility that the detected changes were caused by a random event.

On all the eight sessions transcriptions the MAS and the IDAAP were applied; the CCRT methodology was applied on the first and the last couple of transcribed sessions.

#### Independent Raters

The coders of CCRT and MAS, who were not the same, were independent raters who had received full training on the use of the respective tool. All coders were blind to the client’s therapeutic results and any other information regarding the therapies.

#### Analysis

In order to meet our goal of performing at the same time an outcome, process and process-outcome evaluation of therapies, we focused on some specific parameters evaluated through the instruments we used.

In more detail, to analyze the therapies’ outcome we considered changes in the pervasiveness of the CCRT’s components and the decrease below their threshold for the MMPI-2′s scales: an increase in pervasiveness of CCRT’s functional components – or a decrease in pervasiveness of non-functional ones – is indicative of a good therapy outcome; at the same time, a decrease of MMPI-2′s scales below their threshold (see the threshold values in MMPI’s results paragraph) also corresponds to a good therapy outcome.

With regards to the therapies’ process evaluation, we considered both trends of the MAS’ Self-reflexivity subscales and trends of IDAAP’s WRAD, WRRL, and REF/WRAD covariation scales. Specifically, a positive trend detected in at least one of the MAS’s subscales is an indication of an increase of that specific metacognitive function, therefore distinguishes a good therapy process; similarly, for what concerns the IDAAP methodology, a positive trend of both WRAD and WRRL scales, remaining over their cut-off through all sessions (see the cut-off values in the IDAAP’s results paragraph), together with a negative trend of the REF/WRAD covariation, are a numerical reflection of a good therapeutic process.

Lastly, given that process and outcome as two strictly intertwined features of therapy and, therefore, are not entirely detachable from one another, the jointed analysis of results gathered through all our instruments and methodologies will allow us to perform an overall evaluation of the whole therapies, giving us a comprehensive overview of the interplay between process and outcome throughout sessions.

## Results

We organized this section taking into consideration the three goals of our work: to evaluate the therapies outcomes, to deepen the features of the FP process and to analyze the relation between process and outcome.

### Evaluation of Treatment Change

#### Core Conflictual Relationship Theme

Data gathered through the CCRT methodology are reported in Table 3. Interrater reliability was assessed at the beginning and at the end of treatment. The independent judges showed an agreement of over 87%; concerning the attribution of cluster categories, Cohen’s κ values ranged from 0.90 (excellent) to 0.43 (medium or good) across all the considered sessions (Fleiss and Cohen, 1973; Fleiss, 1981). It was possible to apply the CCRT method only on seven out of ten therapies. In fact, the CCRT methodology foresees the presence of at least ten relational episodes to calculate the conflictual relational theme, and since functional therapies often adopt bodily and touch-based techniques as an alternative to verbal exchanges, the verbal production was not always sufficient for this aim. For the seven analyzable therapies, we considered the changed detected by CCRT positive if at least one of the three components (Wishes, Response from Other and Response from Self) showed an improvement, that is, a decrease in pervasiveness of non-functional clusters and, at the same time, an increase in pervasiveness of more functional ones. In light of these criteria, four patients (patients E, I, O, and U.) showed a CCRT improvement, two (patients Ot and T) showed no changes, and one (patient G) showed a worsening (see Table 3).

**TABLE 3:** CCRT’s data.

PATIENT	FIRST TWO SESSIONS			LAST TWO SESSIONS			EVOLUTION
	COMPONENT	CLUSTER	PERVAS	COMPONENT	CLUSTER	PERVAS	
E	W	To assert self and be independent	0,625	W	To be close and accepting	0,7	POSITIVE
	RO	Rejecting and opposing	0,375	RO	Helpful	0,6	
	RS	Oppose and hurt others	0,375	RS	Respected and accepted	0,7	
		Self-controlled and self-confident	0,375				
		Disappointed and depressed	0,375				
I	W	To be loved and understood	0,45	W	To oppose, hurt and control others	0,29	POSITIVE
					To feel good and comfortable	0,29	
	RO	Rejecting and opposing	1	RO	Rejecting and opposing	0,43	
	RS	Disappointed and depressed	0,73	RS	Self-controlled and self-confident	0,43	
					respected and accepted	0,43	
O	W	To be controlled, hurt and not responsible	0,45	W	To be controlled, hurt and not responsible	0,45	POSITIVE
	RO	Rejecting and opposing	0,55	RO	Rejecting and opposing	0,27	
					Understanding	0,27	
	RS	Anxious and ashamed	0,55	RS	Respected and accepted	0,36	
U	W	To be controlled, hurt and not responsible	0,81	W	To be loved and understood	0,42	POSITIVE
	RO	Rejecting and opposing	0,54	RO	Likes me	0,33	
					Understanding	0,33	
	RS	Disappointed and depressed	0,36	RS	Respected and accepted	0,5	
Ot	W	To assert self and be independent	0,57	W	To be distant and avoid conflicts	0,6	NO IMPROVEMENT
	RO	Rejecting and opposing	0,71	RO	Rejecting and opposing	0,6	
	RS	Disappointed and depressed	0,57	RS	Disappointed and depressed	0,6	
T	W	To feel good and comfortable	0,57	W	To be distant and avoid conflicts	0,38	NO IMPROVEMENT
					To be close and accepting	0,38	
	RO	Rejecting and opposing	0,43	RO	Upset	0,5	
	RS	Helpful	0,29	RS	Helpful	0,44	
G	W	To be close and accepting	0,5	W	To be close and accepting	0,375	NEGATIVE
	RO	Upset	0,33	RO	Rejecting and opposing	0,625	
		Rejecting and opposing	0,33				
	RS	Helpful	0,66	RS	Disappointed and depressed	0,625	

*W, wishes; RO, responses from others; RS, responses of self.*

#### Minnesota Multiphasic Personality Inventory-2

Data gathered through MMPI-2 at all three time points are reported in Table 4. In all three moments in which the MMPI-2 data were collected, we only considered scales whose values were above the cut-off of 65 (that hints the presence of a pathological state and/or of personality disorders traits). The test’s validity analysis was conducted by analyzing the scores obtained in the validity scales L (Lie), F (Frequency), and K (Correction) and in the additional validity scales Fb (Fback), VRIN (Variable Response Inconsistency) and TRIN (True Response Inconsistency).

**TABLE 4:** MMPI’S data at all three time points.

Patient	MMPI-2′s Clinical* and Content** Scales	Initial	Final	Follow -up
E	Sc*	66	65	54
	Mf *	67	64	64
	Hy *	65	64	61
I	Hs *	83	66	71
	D*	70	60	60
	Hy*	74	69	72
	ANX**	83	59	60
	HEA**	77	62	62
O	Hs*	66	52	N.A.
	HEA **	67	54	N.A.
	OBS**	69	64	N.A.
U	D*	70	62	62
	HEA**	74	70	70
	TRT **	73	70	70
F	Hs*	80	62	60
	D*	92	73	64
	DEP **	78	61	53
	HEA**	74	56	58
	ANX**	77	53	51
	SOD**	77	69	74
L	ANX **	65	56	53
	OBS **	70	70	57
Ot.	OBS**	65	65	54
	SOD**	62	67	65
T	Ma *	66	56	N.A.
	Hy*	70	69	N.A.
	ASP **	66	40	N.A.
J	Pa*	68	56	45
	BIZ**	68	45	38
G	Pd*	68	68	68
	ANG**	66	66	66

*Scales were considered if their values were above the cut-off of 65.*

*NA, not available.*

*Clinical Scales: Hs, Hypochondriasis; D, Depression; Hy, Hysteria; Pd, Psychopathic Deviate; Mf, Masculinity–Femininity; Pa, Paranoia; Pt, Psychasthenia; Sc, Schizophrenia; Ma, Hypomania; Si, Social Introversion.

**Content Scales: ANX, Anxiety; FRS, Fears; OBS, Obsessiveness; DEP, Depression; HEA, Health Concerns; BIZ, Bizarre Mentation; ANG, Anger; CYN, Cynicism; ASP, Antisocial Practices; TPA, Type A; LSE, Low Self-Esteem; SOD, Social Discomfort; FAM, Family Problems; WRK, Work Interference; TRT, Negative Treatment Indicators.

All these criteria taken into account, six of the considered psychotherapies (patients E, I, O, U, F, and L) showed a positive trend, three (patients Ot, T, and G) showed a stable/not changing trend for all scales and one (patient J) showed a worsening for at least one scale (see Table 5).

**TABLE 5:** MAS Self-Reflexivity data at 4 timepoints.

Patient	MAS – Self-Reflexivity	T0	T1	T2	T3
E	A3, A4 – Characterization (Yes)	38	34	36	40
	A3, A4 – Characterization (No)	8	2	1	1
	A5, A6 – Differentiation (Yes)	11	10	9	8
	A5, A6 – Differentiation (No)	3	2	2	1
	A7 – Relations between variables (Yes)	6	5	6	5
	A7 – Relations between variables (No)	1	1	0	1
	A8, A9 – Integration (Yes)	6	5	4	5
	A8, A9 – Integration (No)	2	1	2	0
I	A3, A4 – Characterization (Yes)	40	36	41	35
	A3, A4 – Characterization (No)	18	11	7	4
	A5, A6 – Differentiation (Yes)	12	12	9	9
	A5, A6 – Differentiation (No)	1	4	2	1
	A7 – Relations between variables (Yes)	6	4	5	4
	A7 – Relations between variables (No)	1	0	1	0
	A8, A9 – Integration (Yes)	8	6	6	6
	A8, A9 – Integration (No)	2	2	1	1
O	A3, A4 – Characterization (Yes)	41	39	37	34
	A3, A4 – Characterization (No)	9	11	6	3
	A5, A6 – Differentiation (Yes)	14	12	10	11
	A5, A6 – Differentiation (No)	5	4	3	2
	A7 – Relations between variables (Yes)	6	5	6	6
	A7 – Relations between variables (No)	2	2	2	1
	A8, A9 – Integration (Yes)	5	6	7	7
	A8, A9 – Integration (No)	1	2	1	0
U	A3, A4 – Characterization (Yes)	35	29	31	27
	A3, A4 – Characterization (No)	11	13	12	3
	A5, A6 – Differentiation (Yes)	13	8	13	11
	A5, A6 – Differentiation (No)	6	3	3	2
	A7 – Relations between variables (Yes)	5	4	5	4
	A7 – Relations between variables (No)	1	0	1	0
	A8, A9 – Integration (Yes)	5	5	5	5
	A8, A9 – Integration (No)	2	2	1	0
F	A3, A4 – Characterization (Yes)	25	30	32	28
	A3, A4 – Characterization (No)	10	8	12	6
	A5, A6 – Differentiation (Yes)	9	7	14	11
	A5, A6 – Differentiation (No)	3	3	4	2
	A7 – Relations between variables (Yes)	4	5	5	6
	A7 – Relations between variables (No)	2	1	0	1
	A8, A9 – Integration (Yes)	4	4	4	4
	A8, A9 – Integration (No)	0	0	0	0
L	A3, A4 – Characterization (Yes)	31	27	34	21
	A3, A4 – Characterization (No)	12	8	11	10
	A5, A6 – Differentiation (Yes)	11	12	8	8
	A5, A6 – Differentiation (No)	2	3	2	2
	A7 – Relations between variables (Yes)	4	4	5	4
	A7 – Relations between variables (No)	0	1	0	0
	A8, A9 – Integration (Yes)	4	5	7	5
	A8, A9 – Integration (No)	1	0	1	0
Ot	A3, A4 – Characterization (Yes)	29	28	29	21
	A3, A4 – Characterization (No)	4	9	13	7
	A5, A6 – Differentiation (Yes)	16	14	13	12
	A5, A6 – Differentiation (No)	2	3	3	3
	A7 – Relations between variables (Yes)	2	2	4	3
	A7 – Relations between variables (No)	3	2	2	2
	A8, A9 – Integration (Yes)	4	6	6	6
	A8, A9 – Integration (No)	4	2	2	2
T	A3, A4 – Characterization (Yes)	27	28	29	28
	A3, A4 – Characterization (No)	7	5	3	4
	A5, A6 – Differentiation (Yes)	7	7	7	8
	A5, A6 – Differentiation (No)	3	2	2	1
	A7 – Relations between variables (Yes)	4	4	4	3
	A7 – Relations between variables (No)	1	1	2	2
	A8, A9 – Integration (Yes)	6	5	5	5
	A8, A9 – Integration (No)	2	1	2	2
J	A3, A4 – Characterization (Yes)	31	38	29	30
	A3, A4 – Characterization (No)	5	8	9	9
	A5, A6 – Differentiation (Yes)	6	6	4	6
	A5, A6 – Differentiation (No)	1	1	2	4
	A7 – Relations between variables (Yes)	4	3	3	2
	A7 – Relations between variables (No)	2	3	0	4
	A8, A9 – Integration (Yes)	4	3	4	3
	A8, A9 – Integration (No)	4	5	5	4
G	A3, A4 – Characterization (Yes)	30	35	33	34
	A3, A4 – Characterization (No)	2	6	4	4
	A5, A6 – Differentiation (Yes)	8	6	8	11
	A5, A6 – Differentiation (No)	1	1	2	3
	A7 – Relations between variables (Yes)	5	4	4	4
	A7 – Relations between variables (No)	1	1	0	1
	A8, A9 – Integration (Yes)	4	5	6	5
	A8, A9 – Integration (No)	4	2	4	3

*Yes, values of success; No, values of failures.*

The MAS scales that showed more positive change were *characterization* (for five patients: E, F, L, T, and J), *differentiation* (for two patients; F and T), *ability to build relations between variables* (for one patient: F) and *integration* (for three patients: O, F, and Ot); the *characterization* scale showed a negative trend for just one patient (for one patient: J; see Table 5).

#### Italian Discourse Attributes Analysis Program

Data gathered through the IDAAP methodology are reported in Table 6. We considered a positive evolution of the therapeutic processes when we identified, through the application of the IDAAP methodology on sessions transcripts, a positive trend of WRAD and WRRL scales (i.e., their values had an increasing trend along the eight considered sessions and/or they were both over their threshold, respectively 0.5 for WRAD and 0.54 for WRRL) and, at the same time, negative values for REF/WRAD covariation scale. In light of these criteria, eight out of the ten considered psychotherapies (patients E, I, O, U, F, L, Ot, and T) showed a positive trend, by scoring high values, over the cut off of 0.5 for WRAD and 0.54 for WWRL, starting from the first sessions and remaining high in all the other considered sessions. The remaining two therapies (patients J and G), on the contrary, showed values below the cut off and a negative trend along sessions for both WRAD and WWRL, and yielded positive values in the REF/WRAD covariation, therefore displaying a negative trend (see Table 6).

**TABLE 6:** IDAAP data.

Patient	IDAAP’s scale	Session							
		
		1	2	3	4	5	6	7	8
E	WRAD	0,506	0,508	0,507	0,507	0,507	0,507	0,504	0,507
	WRRL	0,548	0,546	0,544	0,547	0,544	0,546	0,548	0,545
	REF/WRAD	−0,074	−0,082	−0,269	−0,151	−0,236	−0,024	−0,051	−0,178
I	WRAD	0,511	0,504	0,505	0,508	0,505	0,505	0,507	0,505
	WRRL	0,547	0,549	0,543	0,544	0,545	0,547	0,543	0,546
	REF/WRAD	−0,085	−0,203	−0,237	−0,080	−0,259	−0,055	−0,130	−0,096
O	WRAD	0,508	0,508	0,505	0,510	0,509	0,507	0,508	0,508
	WRRL	0,548	0,550	0,550	0,546	0,549	0,551	0,548	0,549
	REF/WRAD	−0,075	−0,031	−0,034	−0,088	−0,191	−0,240	−0,017	−0,062
U	WRAD	0,502	0,501	0,500	0,511	0,502	0,506	0,507	0,504
	WRRL	0,549	0,545	0,547	0,546	0,546	0,547	0,544	0,549
	REF/WRAD	−0,027	−0,121	−0,221	−0,071	−0,094	−0,041	−0,120	−0,308
F	WRAD	0,501	0,508	0,504	0,499	0,508	0,504	0,504	0,505
	WRRL	0,547	0,544	0,547	0,548	0,541	0,543	0,548	0,546
	REF/WRAD	−0,573	−0,083	−0,073	−0,336	−0,026	−0,155	0,065	−0,143
L	WRAD	0,501	0,503	0,505	0,503	0,505	0,513	0,502	0,501
	WRRL	0,549	0,548	0,544	0,547	0,548	0,542	0,548	0,548
	REF/WRAD	−0,157	−0,125	−0,032	−0,114	−0,085	−0,112	−0,220	−0,130
Ot	WRAD	0,506	0,508	0,511	0,510	0,507	0,508	0,513	0,510
	WRRL	0,546	0,547	0,545	0,547	0,547	0,547	0,544	0,546
	REF/WRAD	−0,185	−0,142	−0,032	−0,219	−0,078	−0,234	−0,059	−0,198
T	WRAD	0,509	0,502	0,502	0,507	0,506	0,508	0,506	0,504
	WRRL	0,547	0,550	0,552	0,547	0,547	0,541	0,546	0,547
	REF/WRAD	−0,092	−0,258	−0,356	0,009	0,389	−0,604	−0,307	−0,199
J	WRAD	0,505	0,503	0,507	0,500	0,502	0,502	0,496	0,502
	WRRL	0,548	0,548	0,549	0,547	0,545	0,546	0,545	0,548
	REF/WRAD	0,397	0,307	−0,207	−0,114	0,005	0,117	0,015	−0,093
G	WRAD	0,500	0,497	0,503	0,502	0,494	0,499	0,499	0,500
	WRRL	0,545	0,544	0,547	0,543	0,548	0,547	0,546	0,549
	REF/WRAD	−0,281	−0,416	−0,147	−0,230	−0,097	−0,115	−0,189	−0,176

### Process-Outcome Evaluation

Following the research approach that considers process and outcome as different but, at the same time, strictly intertwined aspects, we conducted a process-outcome evaluation using tools as IDAAP and MAS that, albeit designed to evaluate the therapies processes, as matter of fact also evaluate their outcome.

In order to analyze the relationship between the therapies’ outcomes, analyzed by the CCRT and MMPI-2, and the underlying therapeutic processes, detected by the MAS and IDAAP, we organized the data as shown in Table 7. We indicated, for each patient and for each tool, the type of outcome (i.e., positive improvement, deterioration or no changes), and the type of process (i.e., positive trend, negative trend or stationary trend). Moreover, we added the MMPI-2 diagnosis for each patient, since the clinical and content scales are homogeneous item clusters that assess unitary themes and represent a clear communication about personality and clinical problems from the patient to the psychotherapist. There are 10 clinical scales and 15 content scales, measuring different symptom areas and problems that are included in DSM-5 diagnosis (Trull et al., 1995; Klonsky and Bertelson, 2000; Bagby et al., 2005).

**TABLE 7:** Data concerning patients’ overall scores for each used tool.

	Outcome tool			Process tool		
Patient	CCRT	MMPI-2 final	MMPI-2 follow-up	IDAAP	MAS	DSM 5 diagnoses
E	Positive	Positive	Positive	Positive	Positive	BPD
I	Positive	Positive	NI	Positive	Positive	GAD
O	Positive	Positive	NA	Positive	Positive	OCPD
U	Positive	Positive	Positive	Positive	Positive	GAD
F	NA	Positive	Positive	Positive	Positive	GAD
L	NA	Positive	Positive	Positive	Positive	GAD
Ot	NI	Negative	Positive	Positive	Stationary	SAD
T	NI	Positive	NA	Positive	Stationary	BPD
J	NA	Positive	Positive	Negative	Negative	PPD
G	Negative	NI	NI	Negative	Stationary	ASPD

*NA, not available; NI, no improvement; BPD, Borderline Personality Disorder; GAD, Generalized Anxiety Disorder; OCPD, Obsessive Compulsive Personality Disorder; SAD, Social Anxiety Disorder; PPD, Paranoid Personality Disorder; ASPD, Antisocial Personality Disorder.*

## Discussion

The first and wider goal of this work was to conduct an initial explorative and empirical assessment of the outcome of FP, a model that finds its place within psychodynamic psychotherapy treatments. The reason for this goal was that, despite its historical theoretical and technical roots, up until now no empirical research has been made to validate FP, using neither outcomes nor processes empirical tools. We conducted this research considering patients treated in clinical practices, with diagnoses that ranged from generalized anxiety disorder, to social anxiety disorder, to personality disorders (e.g., borderline, paranoid, antisocial and obsessive-compulsive), as defined in DSM-5.

The results, obtained by comparing and integrating data provided by the different empirical tools we used, seem to give indications that FP is effective in a naturalistic context. In fact, six out of ten psychotherapies showed a very good outcome, two showed good improvement, one showed no improvement and one showed a negative outcome. Results concerning the treatments outcomes were examined taking into account the fact that we used several methodologies which are very different from one another. In fact, the MMPI-2 was self-administered by patients, the CCRT and MAS were applied on session transcripts by judges and, lastly, the IDAAP was applied on session transcripts through a computer-aided methodology. In our view, the heterogeneity of these tools limits bias related to the kind of methodology used for the evaluation. For instance, the case of the patient “J,” that could be classified as a positive case if we consider only the MMPI-2 data, appears as not entirely positive if we also consider the features of psychotherapy process analyzed by IDAAP and MAS, that show negative trends and, thus, are not consistent with the encouraging results obtained with the outcome tools.

The MMPI-2 data collected at follow up showed that results seem to be stable over time, despite the concomitant pandemic situation in which the MMPI-2 follow-up was proposed to many patients. A consideration is due regarding the two cases with a negative outcome: as reported in Table 1, these two patients had a DSM-5 diagnosis of paranoid personality disorder and antisocial personality disorder that, as reported in the literature, have a very problematic prognosis (Gabbard, 2014); this may at least partially explain the negative outcome of the treatments.

The second goal of the research was to identify the features of the FP processes that, as mentioned before, are characterized by very specific and peculiar bodily oriented techniques. Our expectation was that this strong focus on body sensations and processes would foster patients’ sudden contact with their emotional experiences.

Consistent with our expectations, IDAAP data show a well-defined feature of clinical sessions: the patient’s ability to use the symbolizing functions is particularly high, and this is true since the initial sessions. This data, as mentioned above, can be read in light of the specificity of FP approach techniques, which are strongly oriented to bring patients’ focus on bodily sensations that, according to Bucci, are based on the subsymbolic elaboration system. The FP approach inverts the flow of information, that in the classical “talking cure” approach goes from words (symbolic elaboration system) to inner sensations (subsymbolic elaboration system) passing through images (symbolic non-verbal elaboration system). In this approach, information stems from body sensations and activity, and this seems to reflect in the particularly high referential activity values we found.

With regard to specific therapeutic processes, we also expected that a positive FP intervention could be reflected in a slow but constant increase in patients’ ability of self-reflexivity, given that metacognitive impairments are usually present within psychopathology. The data collected by MAS gives us interesting information on the therapeutic processes activated by FP; since the construct of reflexive Self can be considered a relational construct (Fonagy, 1991, 1993, 1995; Main, 1991; Fonagy and Target, 1996), it sheds light on the FP techniques from this viewpoint. Most therapies showed an increment of metacognitive capacity of self-reflexivity in all the subscales, even if with preeminence of characterization. As already mentioned when presenting the characterization subscale, the presence of an increase in its values is not always evidence of a positive outcome. It is indeed preferable not to find consistently high values, because they may hint an underlining obsessive use of the cognitive functions. This perspective is particularly useful for patients with anxiety disorders that, as reported in literature (Wells, 2005), who tend to over concentrate on their emotional states without the activation of cognitive aspects. In our patients with anxiety disorders (which constituted half of our sample), this did not happen, thereby demonstrating that the therapeutic couple developed a process that, albeit concentrated on emotional aspects (due to the bodily techniques), did not neglect the metacognitive aspects, but instead allowed their development. These results seem to be consistent with the ones obtained by the IDAAP methodology.

Our third and last goal was to explore the relationship between clinical processes and overall therapeutic outcome, in line with the process-outcome research approach. Specifically, we expected the presence of an overall positive therapeutic outcome if patients’ contact with emotions, fostered by FP techniques, was accompanied by a good level of elaboration of that same emotional experience, and also if an increase in patients’ self-reflexivity could be detected throughout the sessions. The greater the extent to which both of these aspects are present together, the better the expected therapy outcome.

As mentioned before, results from the IDAAP data analysis confirmed how FP techniques foster a sudden contact of patients with their emotions. Nonetheless, as every clinician knows, the patient’s contact with their subsymbolic inner emotions is not necessarily a synonym of “cure.” On the contrary, it could be iatrogenic to force the patient to get in touch with their inner aspects if this happens without the necessary clinical preparation and without the patient being able to recognize and understand the emotional significance of what happened during the session. In this sense, the consistently high values of the WWRL and the negative values of the REF/WRAD covariation attest the patients’ ability to recognize and understand the emotional significance of events; therefore, they may constitute evidence that FP fosters such ability, allowing patients to therapeutically elaborate the clinical material emerging from bodily techniques which, in turn, ultimately leads to a positive outcome.

Another indication of a link between positive processes fostered by FP techniques and good therapy outcomes comes from the analysis of metacognitive abilities: results from the MAS data analysis showed that therapies in which an increase of self-reflexivity in all the subscales could be classified as positive.

Comprehensively, on the basis of data presented above, it seems possible to claim that the specificity of the FP approach, that focuses the clinician’s attention on bodily sensations and includes body-oriented techniques, fosters the development of a psychotherapy process that activates both patients’ emotional and conflictual aspects and their reflexivity since the very beginning. The combination of these virtuous aspects could be related to the prevailing positive outcome we found in our sample. In other words, FP seems to develop a very peculiar psychotherapy process, since it focuses on patient’s body sensations and inner experiences but integrates them within a narrative approach. The obtained changes are discussed inside the therapeutic relationship, where the therapist integrates the experiences reported by the patients and builds with them new meanings and perspectives.

## Conclusion

Although the study we presented seems to give reliable indications concerning the outcome assessment of the FP approach applied on a heterogeneous sample of patients in clinical practice, it is not free of limitations.

First and foremost, given the methodology we used (i.e., multiple-case study) and the sample we considered, we did not apply inferential statistic to assess the change over time of the constructs we assessed; in order to go on with the process of validation of the FP approach, future research ought to overcome this limitation by including larger samples of patients and applying methodologies which grant the possibility to generalize results.

Another limitation regards the collected research data: we adopted several different research tools, and for some of these data was not available. This is due on one hand to the normal obstacle that a researcher can find with patients in clinical practice (for instance, their unavailability to undergo the follow up assessment) and, on the other hand, to the specific features of the FP approach. On this last point, we were faced with a specific feature of the FP technique, i.e., the bodily approach, that tends to privilege techniques that act on a physical level; these can reduce, for some patients, the verbal production, thereby preventing the application of the CCRT, as it was for three of our patients. In future research, this problem might be avoided by applying other kind of research tools, designed to gather non-verbal or para-verbal aspects of the clinical process (see Rocco et al., 2018) and outcome (see Rocco et al., 2021b).

A further aspect which has not been included in this study but might be insightful, from a clinical point of view, if considered in the future, is the analysis of the development of therapeutic alliance, especially for clinical cases in which the therapeutic process does not have a positive outcome (Rocco et al., 2013).

## Data Availability Statement

The raw data supporting the conclusions of this article will be made available by the authors, without undue reservation.

## Ethics Statement

The studies involving human participants were reviewed and approved by researchers of both University of Campania “Luigi Vanvitelli” and University of Padua. Informed consent was obtained from all participants. The study was conducted in accordance with the Helsinki Declaration.

## Author Contributions

All authors listed have made a substantial, direct, and intellectual contribution to the work, and approved it for publication.

## Conflict of Interest

The authors declare that the research was conducted in the absence of any commercial or financial relationships that could be construed as a potential conflict of interest.

## Publisher’s Note

All claims expressed in this article are solely those of the authors and do not necessarily represent those of their affiliated organizations, or those of the publisher, the editors and the reviewers. Any product that may be evaluated in this article, or claim that may be made by its manufacturer, is not guaranteed or endorsed by the publisher.

## Footnotes

1 Within the label “Functional psychotherapy,” we include the therapeutic approach proposed by Luciano Rispoli (1993, 1999, 2008, 2016).
